# Emergency Medicine Residents’ “Just World” Bias Is Not Associated with a Biased Case Mix

**DOI:** 10.5811/westjem.2021.11.53658

**Published:** 2022-01-03

**Authors:** Jessica Edgecomb, Roxana Alexandridis, Benjamin H. Schnapp

**Affiliations:** * University of Wisconsin School of Medicine and Public Health, BerbeeWalsh Department of Emergency Medicine, Madison, Wisconsin; † University of Wisconsin – Madison, Department of Biostatistics, Madison, Wisconsin

## Abstract

**Introduction:**

Belief in a just world is the cognitive bias that “one gets what they deserve.” Stronger belief in a just world for others (BJW-O) has been associated with discrimination against individuals with low socioeconomic status (SES) or poor health status, as they may be perceived to have “deserved” their situation. Emergency medicine (EM) residents have been shown to “cherry pick” patients; in this study we sought to determine whether BJW-O is associated with a biased case mix seen in residency.

**Methods:**

We assessed EM residents on their BJW-O using a scale with previous validity evidence and behavioral correlates. We identified chief complaints that residents may associate with low SES or poor health status, including psychiatric disease, substance use disorder (SUD); and patients with multidisciplinary care plans due to frequent ED visits. We then calculated the percentage of each of these patient types seen by each resident as well as correlations and a multiple linear regression.

**Results:**

38 of 48 (79%) residents completed the BJW-O, representing 98,825 total patient encounters. The median BJW-O score was 3.25 (interquartile range 2.81–3.75). There were no significant correlations observed between BJW-O and the percentage of patients with multidisciplinary care plans who were seen, or patients with psychiatric, SUD, dental or sickle cell chief complaints seen; and a multiple linear regression showed no significant association.

**Conclusion:**

Higher BJW-O scores in EM residents are not significantly associated with a biased case mix of patients seen in residency.

## INTRODUCTION

The emergency department (ED) is often viewed as the gateway to medical care for patients with limited access to resources. Regardless of their ability to pay, anyone who comes through the door is guaranteed a medical screening examination and lifesaving care. However, frequent visits to the ED by patients with less emergent complaints can be perceived as “illegitimate” by clinicians working in already overburdened EDs, leading to stress among healthcare workers and potentially lower quality care for patients.^1^ Physician cognitive biases have previously been demonstrated with regard to which patients are most “deserving” of care,^2^ who will be the most “difficult” to treat effectively,^3^ or a sense of futility in providing additional care.^4^

The belief in a just world (BJW) is a well-studied cognitive bias that “one gets the things that they deserve in life.” When viewed through the lens of one’s own life, this viewpoint can be a protective coping mechanism and is associated with higher rates of satisfaction and fulfillment, and less burnout.^5^ However, when applied to others, it has been associated with discrimination against individuals with low socioeconomic status (SES) or poor health.^6^ There is very limited data on BJW in healthcare. One small study suggested that higher BJW in physicians and nurses was associated with less empathetic feelings toward perinatal mothers.^7^ Another study on undergraduate students showed that students with high BJW were most likely to say that they would help a fictitious patient who was not responsible for their illness.^8^ Evidence is mixed, however, on whether implicit biases uniformly manifest in the clinical environment.^9^,^10^

Previous studies have shown that emergency medicine (EM) residents “cherry pick” the patients they see during their training, selectively choosing specific chief complaints faster than others.^11^ However, this has not been shown to be a universal phenomenon.^12^ In this study we sought to examine whether resident physician BJW for others (BJW-O) is associated with a biased case mix seen during residency training.

## METHODS

This was a cross-sectional, retrospective study that took place at a single, midwestern, academic institution from 2019–2021, and examined patient encounters from 2016–2020. All residents currently in training were eligible, as well as the most recently graduated class (48 total). The clinical site where we conducted the study is a 58-bed, tertiary care facility with approximately 60,000 patient visits annually. The majority of patients evaluated in the ED were White and insured by Medicare/Medicaid, with Black patients making up 11% of visits, Hispanic patients 7%, and Asian patients 3%. Substance use disorder (SUD) encounters are primarily for alcohol and pharmaceutical polypharmacy. Residents are assigned to work in one of three areas, North, staffed by 2–3 residents/advanced practice providers (APPs), including at least one postgraduate year (PGY)-2 or PGY-3 resident, South, staffed by 2–3 residents/APPs, including at least one PGY-3 resident, or pediatrics, staffed with 2–3 residents. For most of the study, clinicians on North or South were able to assign themselves to any adult patient in the department; in mid-2019 pods were created, dividing the responsibility for the ED beds roughly in half. Residents spend 5/13 blocks at the main clinical site during their first year, 6/13 in second year, and 8/13 in their third year.

Two authors (one faculty and one resident EM physician) identified groups of patients who may have been perceived by ED residents as having low SES or poor health status based on a review of the literature. Patients with multidisciplinary care plans, patients with psychiatric, SUD, and dental or sickle cell disease chief complaints were selected as surrogate markers, as patients visiting the ED for these complaints have been shown to be vulnerable to clinician bias and stigma,^1^ and patients who present frequently to the ED have previously been shown to be of lower SES and have significant medical issues.^13^ Patients with multiple ED visits within a short time frame that are felt to be avoidable are flagged by a multidisciplinary team including nursing, EM, primary care, and relevant specialists who develop a care plan that can then be implemented during their visit to ensure consistency. These patients are prominently flagged on the electronic health record track (EHR) board (Epic Systems Corporation, Verona, WI) to ensure that there are no opportunities for these care plans to be missed. Patients with this flag appearing in their chart were used as a proxy for frequent ED visitors, as the population that visits the ED most frequently can be highly variable over time and these patients are not easily identifiable without experience or closely examining the chart.

**Population Health Research Capsule**

What do we already know about this issue?

*Belief in a just world (BJW) has been associated with discrimination against individuals with low socioeconomic status or poor health*.

What was the research question?

*Is resident physician BJW for others (BJW-O) associated with a biased case mix seen during residency training?*

What was the major finding of the study?

*Emergency medicine residents’ BJW-O is not associated with a biased patient case mix seen*.

How does this improve population health?

*BJW does not appear essential to assess as part of resident selection or training to ensure a comprehensive training experience*.

We used the patient’s assigned chief complaint to categorize encounters, as this was the information most likely accessible to residents when assigning themselves to patients. Encounters with a listed chief complaint of “psychiatric problem,” “anxiety,” “depression,” and “suicidal” were included in the analysis as psychiatric encounters. Encounters with a chief complaint of “drug/alcohol issues,” “alcohol intoxication,” and “overdose” were included as SUD encounters.

To create “percentage seen” metrics for patients with complex care plans or with psychiatric and SUD chief complaints we abstracted from the EHR each resident’s number of encounters with patients in each of these categories as the first assigned resident at the residency’s main ED site and then divided by their total number of patients seen in this ED during residency up to that point. We also examined the percentage of shifts worked in the South pod of the ED, as residents were in closer proximity to the rooms generally used for patients with psychiatric chief complaints and may have felt compelled to assign themselves to these patients. Patients received at sign-out were not included in a resident’s total, as residents have less agency in determining which of these patients they are assigned to.

Residents were administered the Belief in a Just World Scale (Appendix A), which measures both BJW for self (BJW-S) and BJW-O. Scores for each scale range from 1 to 5; higher scores represent stronger BJW-O and scores of 3 or lower have been categorized as “low BJW,” while scores of 4 or higher have been categorized as “high BJW.”^7^ Strong validity evidence for BJW in an undergraduate population exists,^14^ and BJW has previously been shown to be stable over time^15^ and to correlate with real-world behavioral outcomes in a general French population.^6^ The instrument was administered to residents via computer-based survey (Qualtrics, Provo, UT) and delivered by email. Participation was fully voluntary. While results could not be kept anonymous due to the need to match with personal encounters data, all responses were kept strictly confidential and stored on password-protected computers.

A multivariable linear regression model was fitted to BJW-O as response variable, with characteristics of interest (percent of patients with multidisciplinary care plans, psychiatric chief complaints, SUD chief complaints) included as predictor variables. We also included in the model the percentage of shifts worked in the South pod as a covariate to adjust for potential confounding. Linear regression assumptions were checked, and all hypothesis testing was two sided, with significance set as *p*-value < 0.05. We calculated Pearson correlations (*r*) between BJW-O and each of the variables of interest, together with 95% confidence intervals for *r* and corresponding *p*-values. A *p*-value <.05 was considered statistically significant. All statistical analyses were performed with R v4.0.3 (The R Project for Statistical Computing, Vienna, Austria).

This study was determined to be exempt quality improvement under the University of Wisconsin-Madison Institutional Review Board guidelines.

## RESULTS

Responses from 38/48 residents (79% response rate: 10 PGY-1, 10 PGY-2, 10 PGY-3, and 8 PGY-4) were available for analysis with no missing data noted for any variable of interest, representing 98,825 total patient encounters with a median of 2,691 patients per resident (interquartile range [IQR] 1,785–3,364). The median BJW-O score was 3.25 (IQR 2.81–3.75). Table 1 summarizes the linear multivariable regression model coefficients. Dental pain and sickle cell disease were dropped from the analysis, as there were too few of these cases per resident.

None of the four predictor variables in the model was found to have a statistically significant impact on BJW-O. The regression model showed a multiple R^2^ value of 0.09883, indicating that 9.88% of the variability observed in the BWJ-O scores could be explained by the four predictors investigated.

From the correlation results shown in Table 2, a nonsignificant small correlation of BJW-O with the percent of patients with a multidisciplinary care plan (*r* = 0.174, *p* = 0.297) and with SUD (*r* = 0.107, *p* = 0.521) was found among the main variables of interest, and small nonsignificant correlations of BJW-O with the auxiliary variables of percent of patients with dental chief complaints (*r* = 0.203, *p* = 0.223), and with BJW-S scores (*r =* 0.098, *p* = 0.56).

## DISCUSSION

The BJW-O scores do not appear to explain the percentage of patients with multidisciplinary care plans or the percentage of patients with psychiatric or SUD chief complaints seen by residents. While residents demonstrated a broader range of BJW-O scores (1–4.9) than previously reported in healthcare providers (2.3–4.7),^7^ this study contrasts with what has been seen in other experimental work on BJW-O;^6^ however, it is consistent with other literature on the impact of implicit biases.^10^

While it is tempting to take the lack of evidence of BJW-O bias affecting residents’ case mix found here as evidence of lack of bias toward these patients, it remains possible that this bias appeared in other ways. This study did not examine the care that was delivered to patients; patients perceived to have poor health status or low SES could have received slower care, lower doses of pain medication, or a less thorough evaluation. The original study on EM resident “cherry-picking” also examined the time elapsed before residents picked up each patient rather than case mix.^11^ It is also possible that the BWJ-O bias manifested in slower pickup times instead of altered case mix.

The lack of association that was found may reflect medical complexity mitigating any potential “selection effect.” For example, a patient with a SUD chief complaint could be a patient with polysubstance overdose requiring intubation, while a patient with a psychiatric chief complaint could be an acute threat to staff requiring chemical restraint. Alternatively, social desirability bias, where residents feel motivated to exhibit their virtuous behavior and altruism to their co-workers may exert a corrective effect against BJW bias and has been postulated in other studies of physician behavior.^16^ This motivation may be especially powerful in EM, where the unofficial motto is, “Anyone, Anything, Anytime.”^17^ Residents also only exert a certain amount of control over their next patient; random chance plays a large role that may have attenuated any potential effects.

For program directors, these results should be encouraging. At this time, it appears that BJW does not need to be assessed as part of resident selection or training to ensure a comprehensive training experience. However, more research should be done to confirm these findings.

## LIMITATIONS

This was a single-center study, conducted with four residency classes, at the primary ED training site. It was conducted at a large, tertiary care academic center that sees a relatively low volume of uninsured and undomiciled patients; it is possible that results would be different in a different medical setting with a different patient population, or with a larger sample size. This was a correlational study; it is possible that other factors not controlled for, such as percentage of night shifts worked, had a larger influence on case mix. Burnout has also been shown to affect BJW,^18^ which could also explain the differences that were found. Additionally, BJW has been shown to vary by race.^19^

For this study we chose chief complaints that the author group felt may be perceived to be associated with low SES or poor health status; it is possible that the groups chosen were not perceived by residents in this way, or that other groups might have been more affected. Patients’ chief complaints also may not have matched their true reason for presentation. Operational changes, such as a switch to a pod system and the COVID-19 pandemic, may have also affected resident case mix in unpredictable ways.

## CONCLUSION

Higher resident BJW-O scores were not correlated with a lower percentage of patients with multi-disciplinary care plans, or psychiatric, SUD, dental or sickle cell chief complaints seen in residency. While the assessment of resident personality traits and their impact on training and patient care is in its infancy, this study suggests that belief in a just world for others does not manifest as a biased case mix.

## Supplementary Information



## Figures and Tables

**Table 1:** Results of multiple linear regression model evaluating the impact of percentage of patients seen with complex care plans, and psychiatric or substance use disorder chief complaints and the percentage of shifts on a resident’s belief in a just world for others.

	Estimate	Std. error	Statistic	*p*-value	Lower 95% CI	Upper 95% CI
Intercept	3.154	2.371	1.33	0.192	−1.669	7.977
Care plan %	0.445	0.313	1.421	0.165	−0.192	1.082
Psych CC %	−0.17	0.2	−0.849	0.402	−0.576	0.237
SUD %	0.405	0.444	0.913	0.368	−0.497	1.307
South %	−0.038	0.035	−1.094	0.282	−0.109	0.033

*CI*, confidence interval; *CC*, chief complaint; *SUD*, substance use disorder.

**Table 2:** Correlations (Pearson’s r) of “belief in a just world for others” scores with belief in a just world for self and the percentage of patients seen with a care plan, psychiatric, substance use disorder, and sickle cell or dental pain chief complaints.

Variable	Mean (SD)	*r*	Lower 95% CI	Upper 95% CI	*p*-value
BJW-O	3.26 (0.85)				
BJW-S	4.73 (0.36)	0.098	−0.229	0.405	0.56
Care plan %	4.95 (0.47)	0.174	−0.155	0.467	0.297
Psych CC %	4.05 (0.73)	−0.076	−0.386	0.25	0.649
SUD CC %	1.40 (0.32)	0.107	−0.22	0.413	0.521
Sickle cell CC %	0.19 (0.11)	0.028	−0.294	0.344	0.868
Dental CC %	0.32 (0.15)	0.203	−0.125	0.49	0.223
South shift %	53.16 (4.09)	−0.157	−0.454	0.171	0.346

*SD*, standard deviation; *CI*, confidence interval; *CC*, chief complaint; *BJW-O*, belief in a just world for others; *BJW-S*, belief in a just world for self; *SUD*, substance use disorder.

## References

[1] Van Nieuwenhuizen A, Henderson C, Kassam A (2013). Emergency department staff views and experiences on diagnostic overshadowing related to people with mental illness. Epid Psychiatr Sci.

[2] Park DB, Berkwitt AK, Tuuri RE (2014). The hateful physician: the role of affect bias in the care of the psychiatric patient in the ED. Am J Emerg Med.

[3] Moukaddam N, Flores A, Matorin A (2017). Difficult patients in the emergency department. Psychiatr Clin North Am.

[4] Schaulis MD, Snoey ER (2001). Three years, a thousand visits: a case study of the ultimate frequent flyer. Ann Emerg Med.

[5] Lipkusa IM, Dalbert C, Siegler IC (1996). The importance of distinguishing the belief in a just world for self versus for others: implications for psychological well-being. Personality and Social Psychology Bulletin.

[6] Bègue L, Charmoillaux M, Cochet J (2008). Altruistic behavior and the bidimensional just world belief. Am J Psychol.

[7] Clyman RI, Roth R, Sniderman S (1980). Does a belief in a ‘just world’ affect health care providers’ reactions to perinatal illness?. Academic Medicine.

[8] Turner DePalma M, Madey SF, Tillman TC (1999). Perceived patient responsibility and belief in a just world affect helping. Basic and Applied Social Psychology.

[9] Maina IW, Belton TD, Ginzberg S (2018). A decade of studying implicit racial/ethnic bias in healthcare providers using the implicit association test. Soc Sci Med.

[10] Dehon E, Weiss N, Jones J (2017). A systematic review of the impact of physician implicit racial bias on clinical decision making. Acad Emerg Med.

[11] Patterson BW, Batt RJ, Wilbanks MD (2016). Cherry picking patients: examining the interval between patient rooming and resident self-assignment. Acad Emerg Med.

[12] Schnapp BH, Fleming E, Kraut AS (2019). Maggots, mucous and monkey meat: Does disgust sensitivity affect case mix seen during residency?. West J Emerg Med.

[13] Byrne M, Murphy AW, Plunkett PK (2003). Frequent attenders to an emergency department: a study of primary health care use, medical profile, and psychosocial characteristics. Ann Emerg Med.

[14] Bègue L, Bastounis M (2003). Two spheres of belief in justice: extensive support for the bidimensional model of belief in a just world. Journal Pers.

[15] Dalbert C (2001). The Justice Motive as a Personal Resource: Dealing with Challenges and Critical Life Events.

[16] Huizinga MM, Cooper LA, Bleich SN (2009). Physician respect for patients with obesity. J Gen Intern Med.

[17] Zink BJ (2005). Anyone, Anything, Anytime: A History of Emergency Medicine.

[18] Desrumaux P, Gillet N, Nicolas C (2018). Direct and indirect effects of belief in a just world and supervisor support on burnout via bullying. Int J Environ Res Public Health.

[19] Hunt MO (2000). Status, religion, and the” belief in a just world”: comparing African Americans, Latinos, and Whites. Soc Sc Q.

