# Supplementary material for: Emergency Medicine Residents’ “Just World” Bias Is Not Associated with a Biased Case Mix
Source: West J Emerg Med. 2022 Jan 3;23(1):95–9. doi: 10.5811/westjem.2021.11.53658 (PMC8782141; doi:10.5811/westjem.2021.11.53658)
Supplement: Supplementary file 1 [file wjem-23-95-s001.pdf]

The following questions pertain to fairness.

In this first set of questions we are interested in your perceptions of fairness with respect to OTHERS. Please mark your level of agreement using the 7-point scale shown below.

1	2	3	4	5	6	7
Strongly Disagree	Disagree	Slightly Disagree	Neutral	Slightly Agree	Agree	Strongly Agree

Your Rating	Statement
	1. I feel that people generally earn the rewards and punishments that they get in this world.
	2. People usually receive the outcomes that they deserve.
	3. People generally deserve the things that they are accorded.
	4. I feel that people usually receive the outcomes that they are due.
	5. People usually use fair procedures in dealing with others.
	6. I feel that people generally use methods that are fair in their evaluations of others.
	7. Regardless of the specific outcomes they receive, people are subjected to fair procedures.
	8. People are generally subjected to processes that are fair.

In this next set of questions we are interested in your perceptions of fairness with respect to YOURSELF. Please mark your level of agreement using the 7-point scale shown below.

1	2	3	4	5	6	7
Strongly Disagree	Disagree	Slightly Disagree	Neutral	Slightly Agree	Agree	Strongly Agree

Your Rating	Statement
	1. I feel that I generally earn the rewards and punishments that I get in this world.
	2. I usually receive the outcomes that I deserve.
	3. I generally deserve the things that I am accorded.
	4. I feel that I usually receive the outcomes that I am due.
	5. People usually use fair procedures in dealing with me.
	6. I feel that people generally use methods that are fair in their evaluations of me.
	7. Regardless of the specific outcomes I receive, I am subjected to fair procedures.
	8. I am generally subjected to processes that are fair.

Scoring: Four lower order subscales may be calculated. **Distributive Justice for Others (DJ-Others)** is the sum or average of the first four items from the justice for others scale, while **Procedural Justice for Others (PJ-Others)** is the sum or average of the last four items from the self justice scale. **Distributive Justice for Self (DJ-Self)** is the sum or average of the first four items from the self justice scale, while **Procedural Justice for Self (PJ-Self)** is the sum or average of the last four items from the self justice scale. Depending on research interests, higher order Self-Other Justice and Procedural-Distributive justice subscales also may be calculated by summing or averaging the appropriate eight lower order subscale items (e.g., Distributive Justice = sum or average of DJ-self and PJ-self items).

Lucas, T., Zhdanova, L., & Alexander, S (2011). Procedural and distributive justice beliefs for self and others: Assessment of a four-factor individual differences model. *Journal of Individual Differences, 32*, 14-25.
